# *QuickStats*: Percentage Distribution* of Adult Day Services Center Participants, by Place of Residence^^† ^^— National Study of Long-Term Care Providers, United States, 2016

**DOI:** 10.15585/mmwr.mm6702a9

**Published:** 2018-01-19

**Figure:**
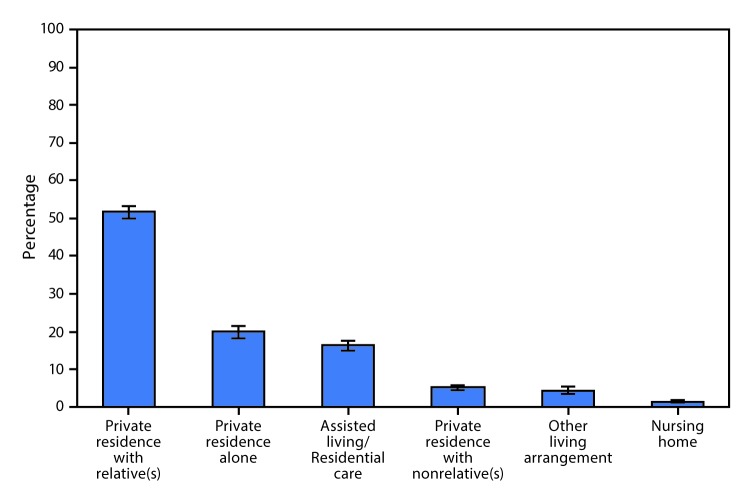
In 2016, 51.5% of adult day services center participants lived in a private residence with relative(s), 19.9% lived alone in a private residence, 16.3% lived in an assisted living/residential care community, 5.3% lived in a private residence with nonrelative(s), 4.5% had another living arrangement, and 1.5% lived in a nursing home.

**Source**: National Study of Long-Term Care Providers, 2016. https://www.cdc.gov/nchs/nsltcp.htm.

